# Quick microbial molecular phenotyping by differential shotgun proteomics

**DOI:** 10.1111/1462-2920.14975

**Published:** 2020-03-11

**Authors:** Duarte Gouveia, Lucia Grenga, Olivier Pible, Jean Armengaud

**Affiliations:** ^1^ Laboratoire Innovations technologiques pour la Détection et le Diagnostic (Li2D) Service de Pharmacologie et Immunoanalyse (SPI) CEA, INRAE, F‐30207 Bagnols‐sur‐Cèze France

## Abstract

Differential shotgun proteomics identifies proteins that discriminate between sets of samples based on differences in abundance. This methodology can be easily applied to study (i) specific microorganisms subjected to a variety of growth or stress conditions or (ii) different microorganisms sampled in the same condition. In microbiology, this comparison is particularly successful because differing microorganism phenotypes are explained by clearly altered abundances of key protein players. The extensive description and quantification of proteins from any given microorganism can be routinely obtained for several conditions within a few days by tandem mass spectrometry. Such protein‐centred microbial molecular phenotyping is rich in information. However, well‐designed experimental strategies, carefully parameterized analytical pipelines, and sound statistical approaches must be applied if the shotgun proteomic data are to be correctly interpreted. This minireview describes these key items for a quick molecular phenotyping based on label‐free quantification shotgun proteomics.

## Introduction

As part of an investigation into how the organism functions, molecular phenotyping of a microorganism consists in the extensive description of its biochemical characteristics, which are shaped by both genetic makeup and environmental influences. Approaches to holistic molecular investigation of microorganisms have changed drastically in recent years. Gene sequencing and annotation of new isolates are now routine in many microbiology laboratories. In addition, large‐scale molecular phenotyping tools such as metabolomics and proteomics are gaining momentum, allowing us to more intimately observe how microbial cells function (Bochner, 2009; Nichols *et al*., 2011). Metabolomic or proteomic results can be exploited to generate novel hypothesis on the metabolic, cellular and biological processes affected upon stimulus or condition change and can be ideally complemented with further assays to validate these hypotheses. With the shotgun proteomic approach, a global cellular metabolic view can be obtained, thus surpassing traditional biochemical assays or western blot detection focused on only one specific protein. Today, a deep proteomics analysis can detect and monitor the abundances of thousands of proteins under different conditions (Armengaud, 2013) and is thus an ideal complement to other phenomics methodologies. Remarkably, the sample required for performing such study is usually easy to obtain (5–10 mg of pelleted cells that should be immediately frozen in liquid nitrogen, biological triplicates) and does not necessitate any additional costly reagent as proteins are stable if kept at −80°C contrary to other techniques such as transcriptomics.

However, for many microbiologists, proteomics may still seem quite difficult to grasp. These difficulties stem in part from the high level of expertise and specific instrumentation required, but also from the wide variety of strategies developed to meet the experimental objectives of different experiments. Indeed, several facets of proteomics can be explained by the various characteristics of proteins. For example, multiple proteoforms may be produced from a single bacterial gene (Bland *et al*., 2014). Their primary structure can be evaluated in terms of translational initiation, peptide signal processing, and protease maturation. In addition, proteins may be post‐translationally modified, dramatically increasing the complexity of the proteome (Dai *et al*., 2017). The nature of any post‐translational modifications and corresponding amino acid locations on the polypeptide can be determined by tandem mass spectrometry. The identity of transient protein partners (interactome), information relating to quaternary structure, topology, thermal stability, production, or even their half‐life can all be established by applying specific proteomic strategies. Localization of distinct proteoforms at the subcellular level, both in the periplasm, or the exoproteome for secreted proteins, can be also assessed. It is thus no wonder that researchers from other fields may feel overwhelmed.

Although proteins may be analysed based on their total molecular masses, most proteomic strategies involve the analysis of protein fragments by tandem mass spectrometry (Swanson and Washburn, 2005). Shotgun proteomics consists in proteolyzing the proteins extracted from a biological sample and analysing the resulting peptides with a high‐resolution tandem mass spectrometer coupled to a reverse‐phase chromatography system. For these types of assays, trypsin is the most commonly used protease, because it generates peptides with an average of 10–12 residues, ending with a basic residue (arginine or lysine). Consequently, these peptides are easily observable by mass spectrometry. In a first MS scan, the mass spectrometer records the mass/charge (*m/z*) ratios of the peptide ions eluting from the chromatography at a given time point. Then, the mass spectrometer selects the most abundant ion and triggers its fragmentation. The mass/charge ratios of the secondary ions are then recorded, generating a specific MS/MS spectrum representative of the amino acid sequence of the peptide. Other peptide ions can be selected for fragmentation and MS/MS measurement before a new cycle of MS is launched. The tandem mass spectrometer provides three elements of information: the molecular mass of each peptide, the molecular mass of their chemical fragments–which is dictated by the amino acid sequence of the corresponding peptide–and the abundance of each peptide. Large‐scale analysis of these peptides can be used to identify and quantify the original proteins, while also establishing their sequence coverage.

Differential shotgun proteomics compares the proteome of a biological entity subjected to varying conditions, or those of multiple closely related microorganisms (e.g. wild type versus mutant) in the same conditions. This approach aims to identify proteins that are present at different levels across these samples. Today, a single nanoLC–MS/MS run on a high‐resolution high‐throughput tandem mass spectrometer can monitor the abundances of more than a 1000 proteins. Therefore, this methodology is increasingly used by microbiologists working with proteomics platforms to perform tandem mass spectrometry measurements. In this minireview, we propose guidelines for a winning strategy for differential shotgun proteomics analyses, specifically to help microbiologists when designing their experiments. We also describe some statistical approaches for use when interpreting shotgun proteomics data.

### Spectral counting, a simple strategy to rapidly assess protein abundances

In shotgun proteomics, several strategies have been devised to monitor the abundances of proteins. The most elaborate strategy involves labelling proteins/peptides with specific chemical reagents, mixing the samples, and performing a single nanoLC–MS/MS run in data‐dependent acquisition mode (DDA), where each peptide is isolated by the mass spectrometer before its fragmentation. The mass spectrometry data from the different samples are distinguished using the specific fragmentation signal corresponding to their respective labels. Although interesting on paper, this approach requires larger amounts of initial biological material, fractionation of the samples to allow a comprehensive survey, and repeated measurements (Choi *et al*., 2008). In addition, unfortunately, the reagents required for labelling are also quite expensive.

Another strategy relies on the comparison of signals from nanoLC–MS/MS runs performed systematically for each sample (Neilson *et al*., 2011). Because of its robust performances, this ‘label‐free’ strategy is currently the most used and can be applied to any sample. The abundance of each peptide is directly related to the intensity of the *m/z* peak measured in MS mode (extracted ion chromatogram, XIC), but this signal depends on the ability of ionization of the compound, or can be deduced from the number of MS/MS spectra assigned to it (spectral counts). This last feature was established as a robust proxy for protein abundance, which is particularly appropriate for use in complex samples where the MS signal may be distorted due to the presence of overlapping ion signals (Liu *et al*., 2004). While peak intensities for quantitation is currently becoming popular, we warn the reader to compare XIC and spectral count approaches for specific set of samples as in microbiology drastic proteomic changes may occur triggering a novel variety of signals in one condition that may interfere with the XIC measurements.

Yet another strategy, data‐independent acquisition mode (DIA), was recently proposed (Ludwig *et al*., 2018), but up to now has mainly been used for human proteomics projects. In DIA, all peptide ions are fragmented simultaneously for a certain *m/z* range established *a priori*; it thus generates complex data and requires strong computing skills to perform iterative searches to identify the less abundant proteins.

Due to the difficulty and expense of the alternatives, most current differential proteomic studies are based on estimating the abundances of proteins by spectral counts. Although this strategy is simple to perform, it requires some care to ensure the necessary depth of the MS/MS analysis: in the setting of the instrument parameters, when selecting the number of replicates, the quality of the protein sequence database to be used for interpreting MS/MS spectra, and the strategy used to infer proteins from peptide sequences. For spectral‐count‐based label‐free quantitation of proteins, the exclusion time for MS/MS acquisition is also an important parameter. Although this time window is frequently increased to maximize the number of peptides to monitor and thus identify low‐abundance proteins, a high value (more than 60 s) may be detrimental to accurate spectral counting of the most abundant proteins, leading to under‐evaluation due to signal saturation. Common good practices in shotgun proteomics (Matthiesen *et al*., 2011; Bereman, 2015) include monitoring LC–MS/MS performance using specific standards, randomization of samples prior to injection to avoid batch effects (Cuklina *et al*., 2020), prioritizing biological replicates over technical or analytical replicates, and verifying reproducibility of replicates. When selecting the database against which the recorded MS/MS spectra will be matched, a well‐annotated genome of the microorganism present in the sample would be the best option, but if not available a draft genome sequence can be a valid alternative (Rubiano‐Labrador *et al*., 2014). Alternatively, the annotated genome from the closest phylogenetic relative of the microorganism, or several such genomes (pan‐proteomics) could also be useful when investigating the proteomes of phylogenetically similar bacterial strains (Broadbent *et al*., 2016). In studies comparing several strains, a common database aggregating individual genome‐derived databases can be fruitfully used to reduce the impact of the individual databases on differential results. Computational tools are essential when interpreting proteomics data and are thus continuously being improved to be more efficient, easily accessible, and user‐friendly. Recent benchmarking studies and reviews should be consulted to select the most appropriate ones. The readers addressed here may not require these programmes as the proteomics platform may give the microbiologist the results in terms of protein identification and label‐free quantification. We have therefore focused this minireview on the statistical assessment of data from label‐free protein measurements.

### Pipeline for robust, rapid molecular phenotyping

**Figure 1:**
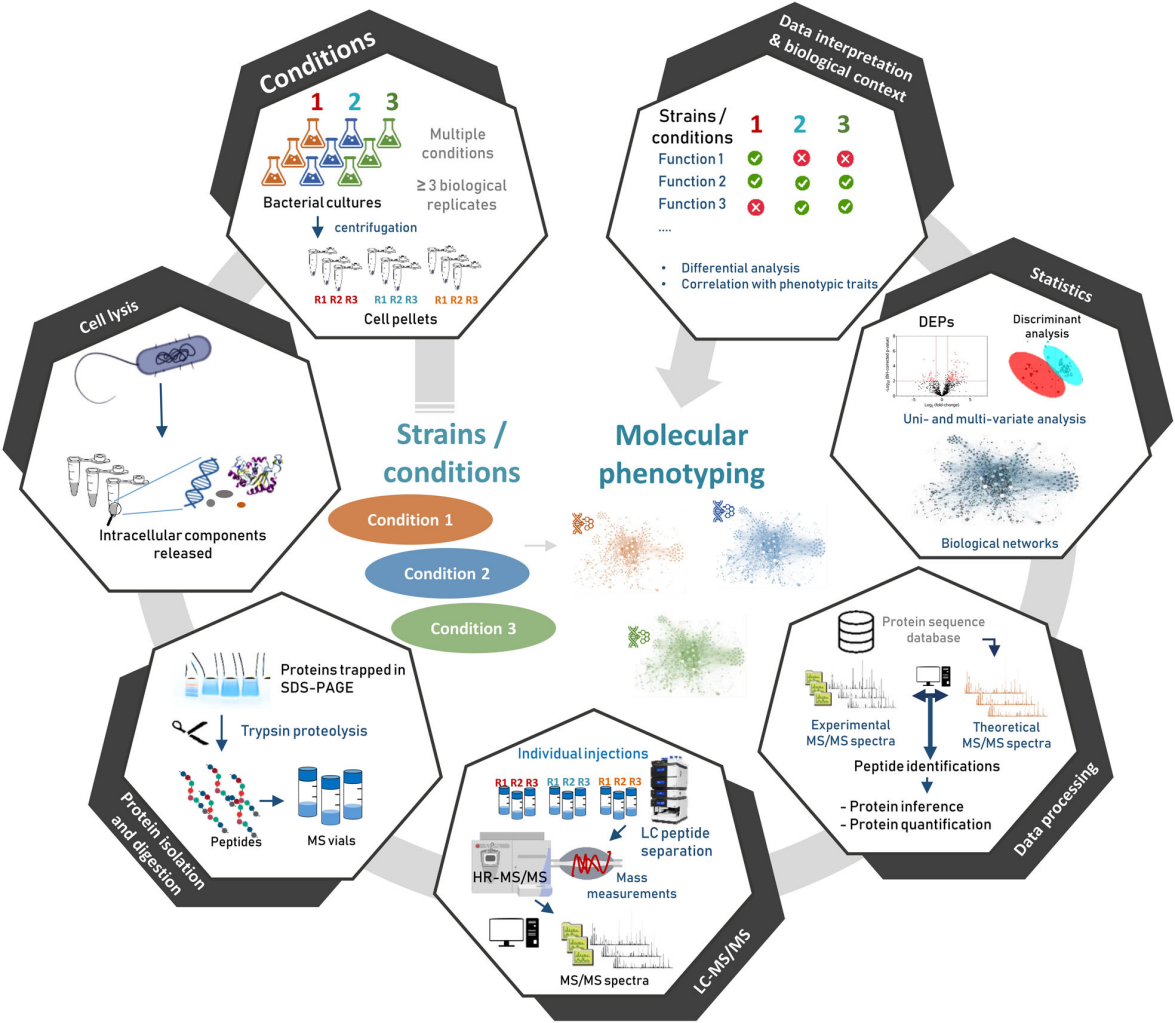
Schematic pipeline for quick but robust molecular phenotyping.

Figure 1 shows the seven steps involved in a quick molecular phenotyping by proteomics. Bacterial cultures or direct sampling are performed in the multiple conditions to be compared. For sure, these conditions should be well documented by preliminary physiological tests (growth curves, biochemistry assays, etc.) to back the proteomics conclusions. A minimum of three biological replicates is recommended, but a higher number should be considered whenever slight differences (typically an increase or decrease of less than 50% of the abundance of key proteins) are expected between conditions or when the biological replicates are found to be highly variable (more than 25% variation for the majority of proteins). Fortunately, in most cases, phenotypic differences in microbiology stem from strong differences in the abundances of the key protein players (Gallois *et al*., 2018). After cell lysis, proteins may be subjected to a short SDS‐PAGE migration to remove any reagent incompatible with mass spectrometry and then proteolyzed in‐gel with trypsin (Hartmann *et al*., 2014). A faster alternative is to perform in‐solution proteolysis directly on the released proteins or after SP3‐purification (Hughes *et al*., 2014; Hayoun *et al*., 2019). However, when using these protocols, particular attention must be paid to detergents included in the lysis buffer, or complex matrices from the initial sample, as they may adversely affect chromatography or tandem mass spectrometry.

Whatever the digestion protocol applied, the resulting peptides are then subjected to LC‐MS/MS and identified. The peptides are then used to establish the list of proteins detected in the whole experiment, i.e. confidently validated in at least one of the samples. Subsequently, the abundance of each protein can be estimated for each replicate and condition based on the intensity of the signals measured for its specific peptides; peptides shared between proteins may also be considered. These data must generally be pre‐processed before performing statistical analysis by means of uni‐ or multi‐variate approaches.

The final and most important step is the interpretation of the results. This step should highlight (i) the proteins for which the abundances vary significantly in a given set of conditions, and if applicable (ii) the aggregated molecular characteristics of proteins, such as metabolic pathways or molecular functional groups, that are modified.

**Figure 2:**
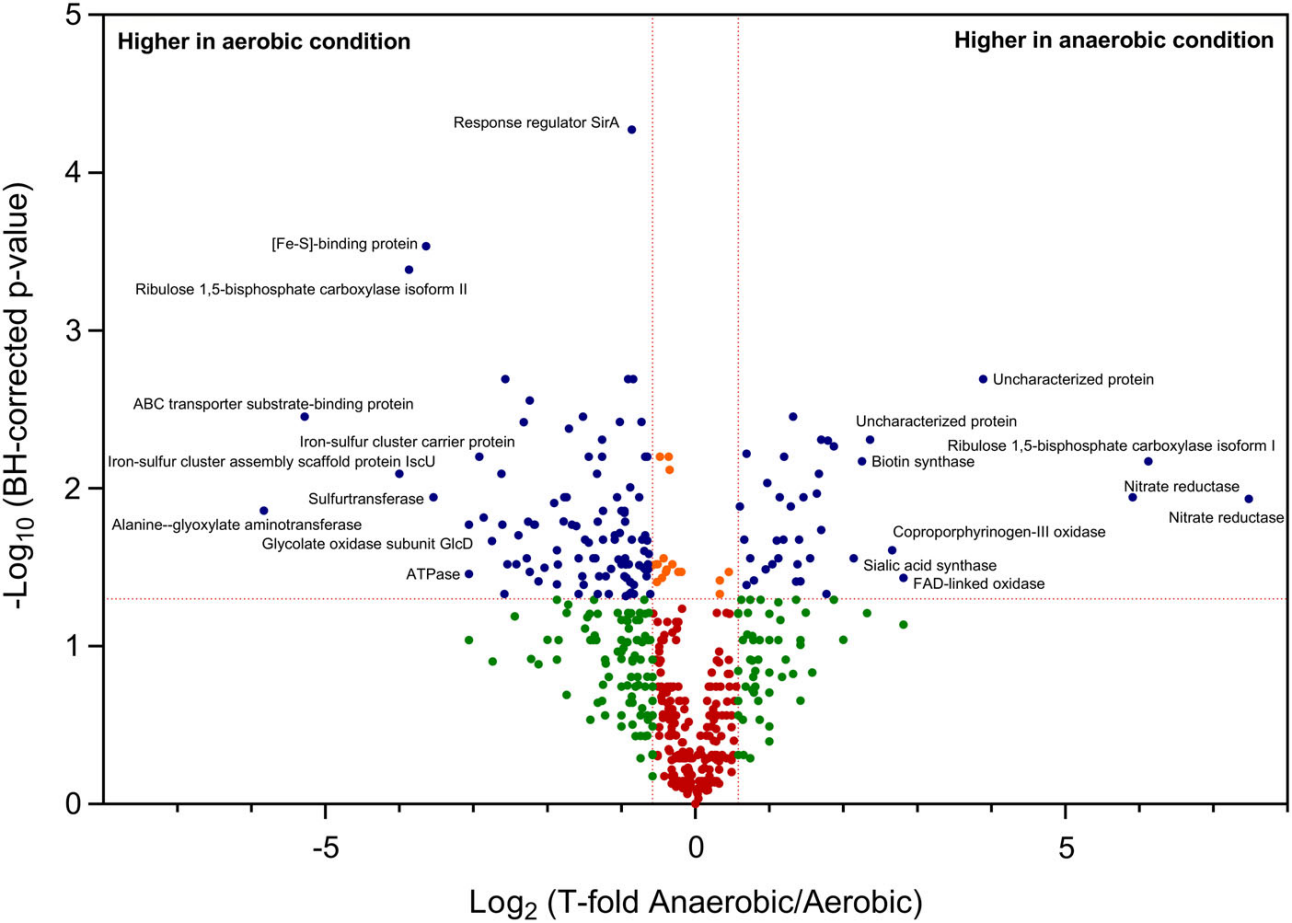
Volcano plot representing univariate analysis of PXD013243 shotgun proteomics data set (anaerobic versus aerobic conditions). The thresholds set were at least 1.5 for the fold change and below 0.05 for the BH‐corrected *P*‐value. Blue dots indicate proteins that satisfy both statistical filters. Orange dots indicate proteins that satisfy only the confidence threshold but have a low fold change. Green dots are proteins that satisfy the fold‐change cut‐off but are not statistically significant. Red dots represent the proteins not satisfying either criterion.

**Figure 3:**
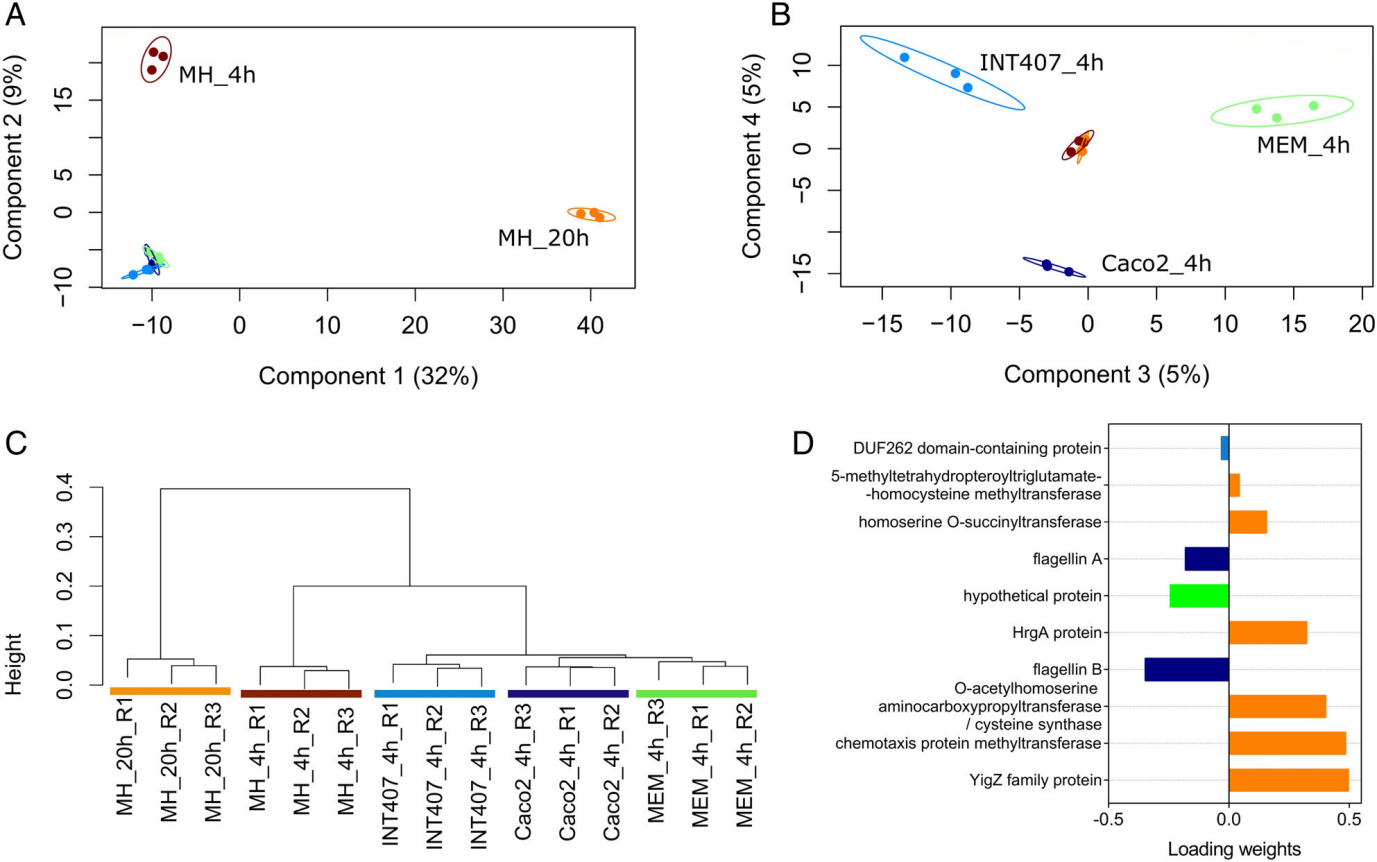
Multivariate analysis of PXD009817 shotgun proteomics data set. A. Sample PLS‐DA plot with 95% confidence ellipses on components 1 and 2. B. Sample PLS‐DA plot with 95% confidence ellipses on components 3 and 4. C. Non‐supervised hierarchical clustering of samples based on the features selected from the sPLS‐DA analysis (subset of 424 proteins). D. Loading plot showing 10 most discriminating proteins on component 1, with colour indicating the condition with a maximal mean abundance value for each protein.

We will use two experiments taken from two recently published microbiological studies to illustrate the statistical tests presented in Figs. 2 and 3. First, Shah *et al*. (Shah *et al*., 2019) described the comparative proteomic analysis of a marine sulfur‐oxidizing isolate, ‘*Candidatus Thioglobus autotrophicus*’, grown under oxic versus anoxic conditions with varying levels of sulfur availability. The authors analysed biological triplicates and identified peptides using a Q‐Exactive HF tandem mass spectrometer following a 145‐min reverse‐phase gradient. MS/MS spectra were acquired with a Top10 strategy, i.e. 10 most abundant precursor ions were sequentially fragmented before performing another full scan, and with a 20‐s dynamic exclusion. The data were deposited on the PRIDE repository under data set identifier PXD013243. They can thus be freely downloaded and exploited. Shah *et al*. showed that the isolate exhibits an unsuspected metabolic flexibility resulting in the production of more organic carbon in the ocean than would have been estimated based solely on their anaerobic phenotype. The second example we use here was published by Negretti *et al*. (Negretti *et al*., 2019), who demonstrated that the *Campylobacter jejuni* bacterium produces the same virulence factors following contact with two different human cell lines. In this case, the authors performed the analysis under five conditions with biological triplicates. The peptides from each sample were monitored with a Q‐Exactive HF tandem mass spectrometer after separation along a 200‐min gradient. The data were also deposited onto the PRIDE repository, under data set identifier PXD009817. The dynamic exclusion was set to 45 s to promote the identification of more peptides from low‐abundance proteins.

### Statistical approaches for spectral count protein quantitation

In a shotgun proteomics experiment, each sample is described by the abundance values of an extensive list of proteins. When specific cellular proteolytic events are suspected, peptide rather than protein intensities may be compared. Whatever the case, data must be pre‐processed if statistical comparisons are to be reliable. A normalization based on the systematic addition of a pseudo spectral count per protein and per sample is recommended (Carvalho *et al*., 2008), avoiding values at zero. When the total numbers of spectral counts are very different between samples, an additional normalization step may be applied (Carvalho *et al*., 2008). In this case, abundances are scaled relative to the sample with the highest spectral count, and the total number of spectral counts detected for each sample is used as a normalizing factor. Alternatively, zero values can be considered as missing values. Several algorithms have been developed to statistically impute missing values in a specific sample based on the existing experimental values of replicates or by comparison to the relative abundance levels of related proteins. The imputation method should be carefully selected (Lazar *et al*., 2016). Following pre‐processing, the spectral count data must be analysed by applying suitable statistical methods dictated by the biological question to be answered. When comparing two conditions (e.g. test condition versus control, mutant versus wild‐type, strain A versus strain B), the comparative analysis consists in calculating a ratio of abundance between the two conditions for each protein. This ratio may be presented as a fold‐change which corresponds to the abundance under condition 1 divided by that measured under condition 2 or as a more sophisticated ratio (Carvalho *et al*., 2008). The statistical significance of this comparison is typically assessed by applying univariate tests to identify the proteins for which differential abundances are detected between the two conditions. Univariate tests examine proteins individually by applying parametric or non‐parametric tests (Student's *t* test, ANOVA, Wilcoxon‐Mann–Whitney test). These tests are hypothesis‐driven and based on rejecting or accepting the null hypothesis within a certain confidence level (a *P*‐value threshold). The confidence level is often set to 0.01 or 0.05, but should be adjusted, and most importantly interpreted, in conjunction with the design and context of the study (Betensky, 2019). *T* test and ANOVAs are commonly used to reveal differences between samples. To be reliable, these tests require that protein abundance for each sample follow a normal distribution, and that sample variances are homogeneous. These aspects can easily be evaluated by measuring the distribution symmetry (skewness) and whether the data are peaked or flat relative to a normal distribution (kurtosis). In proteomic data sets based on spectral counts or XIC area, logarithmic transformations are often required to produce a reasonable normality compatible with statistical rigour. The parametric Student's *t* test is the approach most frequently used to identify proteins presenting statistically significant differences in levels between two samples. It can be based on a minimum of three replicates. The more robust Wilcoxon‐Mann–Whitney non‐parametric test is more recommended but normally requires a higher number of replicates. In large proteomics data sets, because many proteins are quantitated, it is also important to pay attention to the multiplicity of tests performed. Indeed, the repetition of individual tests on thousands of proteins increases the occurrence of type I errors, i.e. false positive results (chance rejection of the null hypothesis when it was true). The false discovery rate (FDR) associated with the repetition of such tests can be evaluated. Multiple testing corrections include the ultra‐conservative Bonferroni procedure or the less conservative Benjamini–Hochberg calculation. The latter is the most popular approach since it limits type I errors without being too conservative, thus also limiting type II errors (false negatives). This calculation aims to control the FDR, i.e. the expected proportion of errors among the rejected hypotheses. By adjusting the list of raw *P*‐values to correspond to an FDR of 0.01, a maximum of 1% of the resulting discoveries are expected to correspond to false positive results. As an example, Fig. 2 shows a volcano plot resulting from a univariate analysis on the PXD013243 data set. We wished to discover the proteins displaying the greatest differential abundance between the two conditions and selected a threshold of at least 50% change (fold of 1.5). The plot shows the distribution of the proteins identified according to log_2_(fold‐change) on the abscissa (x) and statistical confidence on the ordinate (y). The data set contains three biological replicates from two conditions (anaerobic versus aerobic). The mean normalized protein abundance values for each condition were compared by applying multiple *t* tests. The resulting raw *P*‐values were adjusted to an FDR of 0.05 using the Benjamini‐Hochberg method.

Using these parameters, a total of 152 proteins are significantly detected in this data set when comparing the two conditions. The proteins of interest are indicated by the blue dots in the volcano plot (Fig. 2). Among these, 40 are more abundant in the anaerobic condition and 112 are more abundant in the aerobic condition. As indicated in the plot, the most extensive significant changes are for the alanine‐glyoxylate aminotransferase (more abundant in the aerobic condition) and the nitrate reductase (more abundant in the anaerobic condition). We also highlighted the presence of two isoforms of the large subunit of ribulose 1,5‐bisphophoate carboxylase (Rubisco), each being specific of a condition.

For more complex experimental designs, e.g. including a larger number of conditions, classic univariate tests will be more difficult to interpret. The high dimensionality of the data imposes a need for simplification to maximize the relevant features and minimize redundancy. In these cases, multivariate tests that consider whole groups of features together and present a view of the overall structure of the data, are essential to reduce data dimensionality through the selection or extraction of relevant subsets of features – here, proteins (Meng *et al*., 2016). In proteomics, the most commonly used multivariate methods are principal component analysis (PCA), linear discriminant analysis (LDA), partial least squares discriminant analysis (PLS‐DA), and hierarchical clustering (HC).

PCA and HC are both non‐supervised exploratory methods. These methods are non‐supervised as they only take as input the abundance values of proteins per sample, with no need to provide information about the nature of the samples. PCA and HC can be used to classify samples into groups and thus verify the overall homogeneity level between replicates. PCA reduces the dimensionality of the data set by projecting features onto a lower dimensional space, clearly revealing the variability in the data set. HC aims to cluster similar entities together into the same class, through the iterative construction of a hierarchical tree known as a dendrogram.

In contrast, LDA and PLS‐DA are supervised multivariate methodsthe input data are labelled with information about the samples. The two methods are conceptually similar, but PLS‐DA can readily handle multiple dependent categorical variables and is compatible with very noisy data sets, whereas multicollinearity issues may weaken LDA application (Noes & Mevik, 2001). In PLS‐DA, new components maximize the covariance between features (proteins). By using the information provided about the nature of the samples, the new components can specifically be used to discriminate between the sample groups.

PCA, LDA and PLS‐DA can all be used to extract the features (proteins) that contribute to distinguishing sample groups, such as, for example, strains with different molecular profiles and consequently different phenotypes, or conditions producing the most contrasted effects. Figure 3 shows an example of multivariate analysis applied to the data from the PX009817 data set, which correspond to the results of three biological replicates from five different conditions. When merging the results of these 15 samples, a total of 1333 proteins (features) were validated and quantified, resulting in a table with 19 995 measured values. Because of this high dimensionality, a PLS‐DA was performed to reveal the protein subsets discriminating between the different conditions (Fig. 3). The two first main components explain 32% and 9% of the variability. Components 1 and 2 clearly discriminate conditions MH_20h and MH_4h from the other three conditions (Fig. 3A). Components 3 and 4 are more discrete (5% each) but allow the three other conditions to be distinguished (MEM_4h on component 3, Caco2_4h on component 4, INT407 in both components), as visible in Fig. 3B. The subset of proteins that best discriminates the different conditions was then extracted from the four components using a sparse version of PLS‐DA analysis, sPLS‐DA (Le Cao *et al*., 2008), available in the mixOmics R package (Rohart *et al*., 2017). To validate that this subset discriminates the five groups of samples, these 424 proteins were used to build a dendrogram based on correlation‐base distances. Briefly, the distance between samples was determined by the Spearman correlations of their overall protein abundance (i.e. the rankings of the proteins listed as a function of their abundances for all samples were compared). If the features of two samples were highly correlated–if the proteins had almost similar abundance ratios and were ranked similarly – samples would cluster together. As shown in Fig. 3C, five clusters corresponding to the five study conditions were obtained. This subset of proteins can be further explored to verify which proteins correlated with which group of samples, providing invaluable information on the specific bacterial functionalities in each condition. Figure 3D, for example, shows 10 proteins that contribute the most to group discrimination when examining component one, and in which condition they are more abundant. Six of these proteins are more abundant in the MH_20h condition, providing information about the functionalities that discriminate this condition from the others.

This statistical exploration of label‐free quantification data can be performed with a myriad of different tools that cover different levels of expertise of the end‐user (Tsiamis *et al*., 2019). Packages such as RforProteomics (Gatto and Christoforou, 2014) or mixOmics (Rohart *et al*., 2017) are available in R for deep data exploration, statistics and visualization. Non‐expert users can rely on more user‐friendly platforms such as OmicsPlayground (Akhmedov *et al*., 2019), PatternLab (Carvalho *et al*., 2016), Perseus (Tyanova *et al*., 2016), or LFQ‐Analyst (Shah *et al*., 2020) for evaluating differential protein abundances and generating publication‐quality data plots. Functional enrichment of related proteins may be evaluated by clustering based on KEGG features – using GhostKOALA (Kanehisa *et al*., 2016) – or Gene Ontology features. These features can be statistically assessed, compared with the observed phenotypes, and further validated during a new round of directed experiments (see Clair *et al*., 2012 as an example). Thus, the great amount of data generated by shotgun proteomics approaches can be straightforwardly mined using modern statistical tools to extract key information and therefore focus on the notable differences between conditions.

## Concluding remarks

The label‐free shotgun proteomics approach briefly presented here is a robust and rapid procedure compatible with molecular phenotyping of microorganisms in a range of conditions. Although not as accurate as the most exquisite targeted quantitation strategies, spectral counting allows a simple and robust estimation of protein abundance changes when comparing conditions for microbiological samples. In terms of time‐frame, the analysis of the whole proteome from a microorganism grown under three different conditions with four biological replicates per condition can be performed in routine mode with the above‐described methodology within a week: a single working day would be necessary for protein extraction from the 12 samples and trypsin proteolysis, a whole day for the 12 LC–MS/MS runs, interpretation of the raw MS/MS results would also take 1 day, and 2 additional days would be needed for data verification and statistical analysis. The methodology described here is thus easily applicable to tens of conditions and to numerous biological replicates if subtle changes are sought. With results for more than a 1000 proteins in hand, the most important challenge becomes the bibliographic work to be done on the proteins, to determine the enriched functions and pathways revealed by the differential proteomics approach. This information must then be integrated within the specific biological context of the experimental set‐up, and the most interesting discoveries validated by appropriate experiments.
